# Insights into the Corrosion Behavior of Pure Magnesium and Magnesium–Calcium Alloy (Mg-1.8 at.% Ca) in Thin-Film and Bulk Forms

**DOI:** 10.3390/ma18071416

**Published:** 2025-03-23

**Authors:** Hüseyin Zengin, Andrei Ionut Mardare, Andreas Greul, Manuel Hofinger, Gianina Popescu-Pelin, Gabriel Socol, Achim Walter Hassel

**Affiliations:** 1 Institute of Chemical Technology of Inorganic Materials (TIM), Johannes Kepler University Linz, Altenberger Str. 69, 4040 Linz, Austria; andrei.mardare@jku.at (A.I.M.);; 2 Lasers Department, National Institute for Lasers, Plasma and Radiation Physics, Magurele, 077125 Ilfov, Romania; 3 Faculty of Medicine and Dentistry, Department Physics and Chemistry of Materials, Danube Private University, Steiner Landstraße 124, 3500 Krems an der Donau, Austria

**Keywords:** magnesium alloy, thin film, microstructure, corrosion, electrochemistry

## Abstract

This study investigates the microstructural and corrosion properties of pure magnesium (Mg) and Mg-1.8Ca (at.%) alloy in both bulk and thin-film forms. Microstructure investigations showed that the addition of calcium (Ca) to Mg resulted in significant differences in microstructures. The bulk pure Mg exhibited coarse and elongated α-Mg grains, which were refined by Ca addition, together with the formation of a Mg_2_Ca intermetallic phase distributed throughout the microstructure. In contrast, thin-film Mg-1.8Ca alloys displayed a refined single-phase microstructure with uniform nm-scale grains and no intermetallic formation. The electrochemical corrosion tests revealed that the bulk and thin-film pure Mg exhibited comparable corrosion rates, while a substantial difference between the corrosion resistance of bulk and thin-film Mg-1.8Ca (at.%) alloy was observed. The thin-film Mg-1.8Ca (at.%) alloy showed an exceptionally better corrosion resistance, attributed to the formation of a more stable surface film and the absence of a less noble Mg_2_Ca intermetallic phase, ensuring a single-phase microstructure. This study highlights the importance of different manufacturing techniques and microstructural control in improving the performance of Mg alloys for high-tech applications.

## 1. Introduction

Over the last few years, Mg has been identified as one of the most promising material candidates for emerging high-tech applications such as aerospace components, biodegradable implants and highly specialized battery technologies [1,2]. While its properties, including specific strength, damping capacity and biocompatibility, are advantageous, there are still challenges that have to be overcome. For example, Mg and its alloys typically exhibit high corrosion rates, which must be precisely adjustable for their use as structural metals and biodegradable implants [3]. The most common approach to address this challenge is alloying Mg with certain metals [4,5]. Because most alloy properties are highly composition-dependent, identifying the ideal ratios for a certain application usually requires the production of a large number of alloys and performing tedious testing procedures.

One approach to streamlining this procedure is the fabrication of compositional thin-film libraries, where a compositional gradient of two or more metals is deposited onto a substrate, such as silicon (Si) or thermally oxidized Si. These films can subsequently be analyzed with a fine compositional resolution. This method allows fast combinatorial screening of alloys for desired properties. Previous studies already demonstrated the advantages of this method for different alloy systems [6,7,8,9]. In the case of Mg, it was possible to identify Mg-Ca alloys with improved corrosion resistance [10]. Nevertheless, the degree to which the observed properties of thin-film alloys can be translated to bulk materials remains strongly related to the specific material system. Because the thermodynamic and kinetic conditions in thermally evaporated thin films differ from those in bulk materials, direct translatability is not guaranteed. For instance, in this compositional thin-film library, intermetallic-phase formation was not observed. To explore these differences, this study focuses on a certain alloy composition of Mg-Ca (namely 1.8 at.% Ca) which exhibited improved corrosion properties as a thin film. This film has been characterized by physical and electrochemical methods and was compared to the equivalent bulk alloy. The goal was to identify which parameters are directly comparable and in which parameters the translatability is not given. Insights into these relations can lead to vastly accelerated research processes in various fields. In this particular case, it can be used for the development of finely tuned Mg-Ca alloys for structural, battery and implant applications. Additionally, studying the unique properties of thin films separately can reveal potential applications beyond those with bulk alloys. Until now studies focused on Mg-Ca thin films remain very limited [10,11,12]. The gathered insights from this study can provide valuable information for the possible use of these alloys as coatings or films. For example, research on applications like micro batteries designed for implantable devices could benefit greatly from carefully engineered thin films.

Overall, the results demonstrate the usefulness of this combinatorial approach to alloy development. It was possible to show that alloying Mg with 1.8 at.% Ca enhances corrosion resistance, and insights into the translatability of thin-film properties to bulk materials were gathered. Furthermore, unique properties of Mg-Ca thin films were explored which can be beneficial for future research and design of such alloys.

## 2. Materials and Methods

The bulk pure Mg and a Mg-1.8Ca (at.%) alloy were produced via permanent mold casting. First, the pure Mg chips (>99.9%) were melted at 750 °C in a stainless-steel crucible using an electric resistance furnace. Then, pure Ca (>99.9%) chips were added to the molten metal. A mechanical stirring was applied to the mixture for 10 min to ensure compositional homogeneity. After that, the molten metal was poured into a steel mold preheated to 250 °C. Throughout the melting and pouring processes, a gas mixture of CO_2_ and 1% SF_6_ was constantly supplied to the surface of the molten metal. In total, 150 g of metal for each sample was cast into a Y-shaped steel mold. Since the 1.8 at.% Ca refers to 2.93 wt.% Ca, the required proportions of Mg and Ca chips were approximately 145.6 g of Mg and 4.4 g of Ca.

The thin-film samples were fabricated by thermal co-evaporation onto borosilicate glass substrates from pure Mg and Ca sources, which were placed separately in W thermal boats. The rates of deposition were simultaneously monitored through individual quartz crystal microbalances (QCMs) facing each evaporation source. The power for Ca was set between 78 and 85 W, with a deposition rate of 0.01 nm/s. The power for Mg was set at 75 W, with a deposition rate of 1.4 nm/s. Their values were adjusted in real time, using in-house-developed LabView PID software (2020) to adjust the power for each source based on the desired evaporation rate. The base pressure of the evaporation chamber was 1 × 10^−4^ Pa, and the evaporation proceeded until a film thickness of approximately 300 nm was obtained.

The chemical compositions of both bulk and thin-film samples were determined by inductively coupled plasma–optical emission spectroscopy (ICP-OES). The samples were digested using 3% nitric acid (HNO_3_) before analysis. The constituent phases were analyzed by the X-ray diffraction (XRD) method, using an X’Pert Pro MPD system (Malvern Panalytical, Malvern, UK), with CuKα (λ = 154.18 pm) radiation. The scan range was set from 10° to 90° 2θ, with a step size of 2 s per step, and the operating conditions were 45 kV and 40 mA. The microstructural characterizations were performed by scanning electron microscopy using Apreo 2 SEM (Thermo Fisher Scientific, Waltham, MA, USA). Samples were mechanically ground and polished using silicon carbide papers up to 4000 grit, followed by fine polishing with oxide polishing suspension. Cross-sectional SEM images were obtained using focused ion beam (FIB) milling. Additionally, the surface topographies of the corroded and uncorroded samples were revealed by atomic force microscopy (AFM) using a NanoSurf easyScan 2 system in contact mode, using ContAl-G cantilevers with an interaction force of 10 nN at room temperature.

The electrochemical corrosion tests, including open circuit potential (OCP), potentiodynamic polarization (PDP) and electrochemical impedance spectroscopy (EIS) analyses, for the bulk and thin-film samples were conducted using an Ivium Vertex potentiostat (Ivium Technologies, Eindhoven, The Netherlands). A self-designed three-electrode setup was used, consisting of Ag|AgCl|3 M KCl as the reference electrode, Pt wire as the counter electrode and the sample as the working electrode, attached to the side of the cell and forced against sealing from the back side, with a diameter of 5 mm to ensure that only thin film was exposed to the electrolyte. A scan rate of 1 mV s-1 and a voltage range from −0.3 to +0.3 V (vs. OCP) were used to obtain polarization curves. The corrosion current density (i_corr_) values were determined by the intersection of the linear fit, initiated approximately 100 mV below the corrosion potential (E_corr_), with the vertical line extending from E_corr_. The frequency range applied for the EIS measurements was from 100 kHz to 0.1 Hz at OCP, using a sinusoidal voltage amplitude of 10 mV rms. All measurements were carried out in 0.9% NaCl solution at 25 °C and repeated at least three times for each sample.

## 3. Results

### 3.1. Microstructure

**Figure 1:**
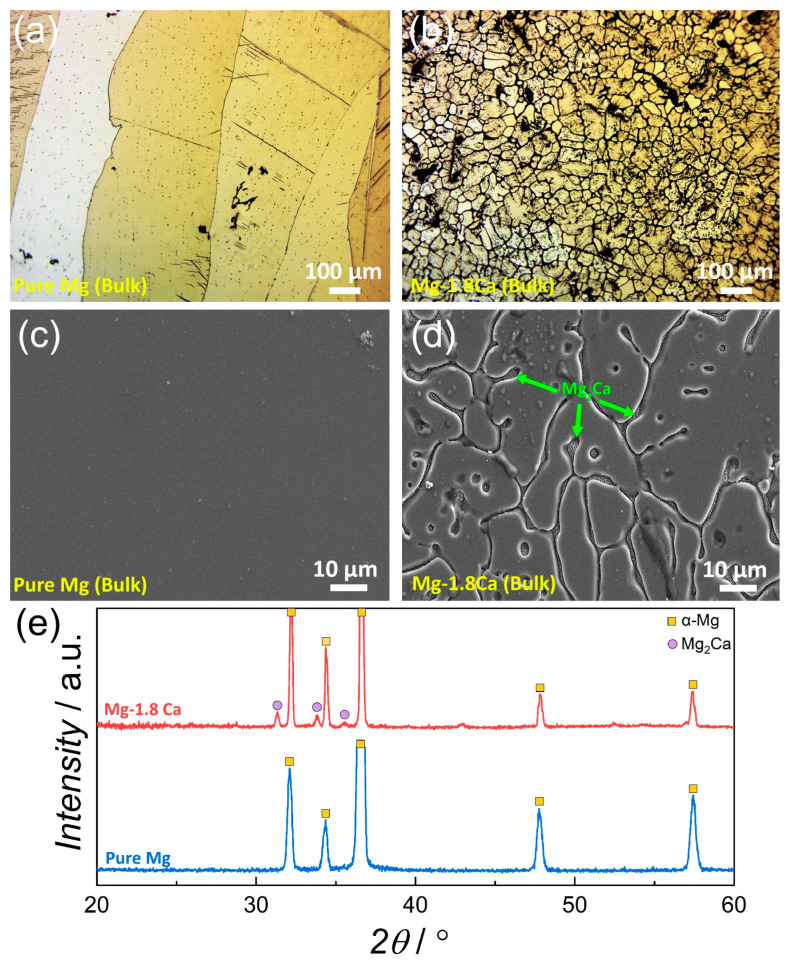
(**a**,**b**) Optical and (**c**,**d**) SEM micrographs of the bulk (**a**,**c**) pure Mg and (**b**,**d**) Mg-1.8Ca (at.%) alloy. (**e**) XRD patterns of the bulk samples.

Figure 1 shows the micrographs and the XRD patterns of the bulk pure Mg and Mg 1.8Ca (at.%) alloy. The optical micrographs of the bulk samples revealed the as-cast grain structure effectively. The pure Mg consisted of large and elongated α-Mg grains, indicating the presence of columnar grain morphology. The average grain size measured perpendicular to the columns was 359 ± 67 µm, whereas it was found to be 1258 ± 183 µm along the column length. It is clear that the addition of 1.8 at.% Ca to pure Mg resulted in a dramatic change in the microstructure. Specifically, the coarse α-Mg grains present in pure Mg showed a significant refinement after the addition of Ca with clearer dendrite arms. The average grain size in the Mg-1.8Ca (at.%) alloy was determined to be 34 ± 4.6 µm. This efficient grain refinement can be mainly attributed to the constitutional undercooling within the diffusion layer ahead of the solid/liquid interface, which was caused by the addition of Ca with low solubility in Mg. This undercooling slows down the diffusion of solutes, thus restricting the crystal growth rate [13,14]. Furthermore, the single-phase microstructure in pure Mg was also replaced with a dual-phase formation in which an intermetallic phase precipitated mostly along the grain boundaries, covering the entire microstructure.

The XRD result in Figure 1e demonstrates that this intermetallic phase in the Mg-1.8Ca alloy was Mg_2_Ca, showing peaks at 31.33°, 33.85° and 35.59° (JCPDS #13-0450). Mg_2_Ca intermetallic phases can typically be seen in Mg-Ca binary alloys [15,16]. The formation of Mg_2_Ca was indeed already expected since it forms in the cast microstructure when the Ca ratio in Mg exceeds its solubility limit of 0.82 at.% (or 1.34 wt.%) according to the Mg-Ca binary phase diagram [17]. It has also been reported in many studies that Mg_2_Ca can precipitate even at much lower Ca ratios, due to the strong segregation effect of Ca in Mg and the non-equilibrium solidification conditions during production [13,18,19,20]. The binary Mg_2_Ca intermetallic phase could also have contributed to the grain refinement by inhibiting the grain growth. Furthermore, the major diffraction peaks for α-Mg in pure Mg sample were observed at 32.31°, 34.36°, 36.66°, 47.76° and 57.44°, where the Mg-1.8Ca alloy displayed α-Mg peaks at 32.21°, 34.37°, 36.63°, 47.81° and 57.35°, correlating well with the (100), (002), (101), (102) and (110) crystal planes, respectively (JCPDS #35-0821). The lattice constants of the bulk and thin-film samples together with the *c*/*a* ratios were calculated, and the values are given in Table 1. It can be seen that the lattice constants of the bulk samples showed slight increases in comparison with the standard values. Even though the *a* and *c* constants of bulk Mg-1.8Ca alloy were lower than those of the bulk pure Mg, the *c*/*a* ratio of pure Mg slightly increased as a result of Ca addition.

**Figure 2:**
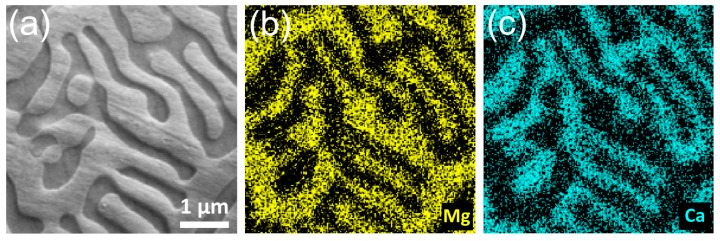
Elemental mapping analyses of the bulk Mg-1.8Ca (at.%) alloy, including (**a**) SEM micrograph of the analyzed area and distributions of (**b**) Mg and (**c**) Ca elements.

The EDS elemental mapping analysis of the bulk Mg-1.8Ca (at.%) alloy is illustrated in Figure 2. The distribution of Mg and Ca elements in the given lamellar eutectic region revealed that the light phases mainly consisted of Mg, whereas the dark phases were enriched with Ca. This suggested that the light-colored regions represented the α-Mg matrix phase, and the dark-colored regions corresponded to the Mg_2_Ca intermetallic phase.

**Figure 3:**
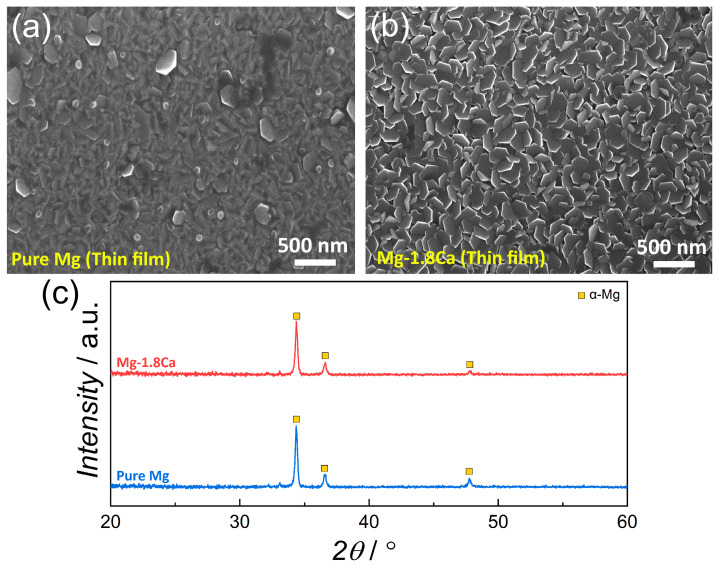
SEM micrographs of the thin-film (**a**) pure Mg and (**b**) Mg-1.8 Ca (at.%) alloy. (**c**) XRD patterns of the thin-film samples.

**Figure 4:**
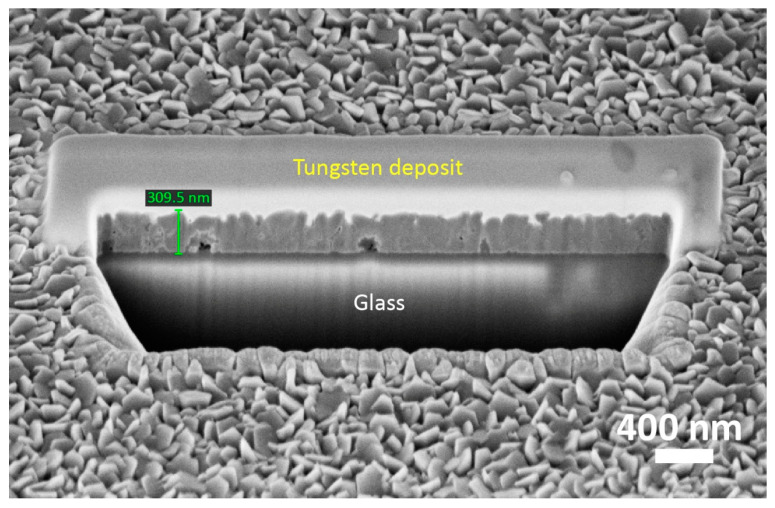
The cross-sectional micrograph of the Mg-1.8 Ca (at.%) thin film, following FIB milling and deposition of tungsten.

The SEM micrographs and the XRD patterns of the thin-film pure Mg and Mg-1.8Ca (at.%) alloys are illustrated in Figure 3. The plan view of the surface of the pure Mg indicated that the film deposition mostly produced a dense structure. However, large and small distinct hexagonal facets occasionally formed as well, corresponding to the termination of film growth with (002) basal plane of Mg having a hexagonal close-packed (HCP) structure. The addition of Ca resulted in the formation of more uniform and equiaxed facets with an average grain size of 97 ± 11.4 nm. Moreover, there was no sign of the presence of any secondary phase within the structure. In addition, the XRD result in Figure 3c showed that there were only crystalline α-Mg peaks in both thin-film samples, with no evidence of the Mg_2_Ca intermetallic phase that was observed in the bulk Mg 1.8Ca (at.%) alloy. This phenomenon in thin films can be primarily attributed to the extremely restricted diffusion of atoms, which immediately freeze upon condensation on the surface of the substrate kept at room temperature during deposition. In certain cases, this may result in the formation of phases or symmetries that are absent in bulk versions of the same alloys [10,21]. Furthermore, it can be seen from Table 1 that the lattice constants of the thin-film samples showed decreases when compared to the bulk samples and the standard values, suggesting a lattice contraction in thin films. This might be due to the quantum size effects and/or the compressive residual stresses to which the thin films are likely exposed at a high rate [22,23]. The cross-sectional SEM micrograph taken from the thin-film Mg-1.8 Ca (at.%) alloy is given in Figure 4. The average thickness of the deposited film was around 309.5 nm. The deposition of thin film was generally observed to exhibit homogeneous and equiaxial-like growth in all directions other than columnar growth, without any characteristic feature. In addition, porosity or impurity-like defects were generally observed at the glass substrate and film interfaces. Since these were very close to the substrate surface, it was thought that they might have originated from residues on the substrate surface before deposition.

### 3.2. Corrosion

**Figure 5:**
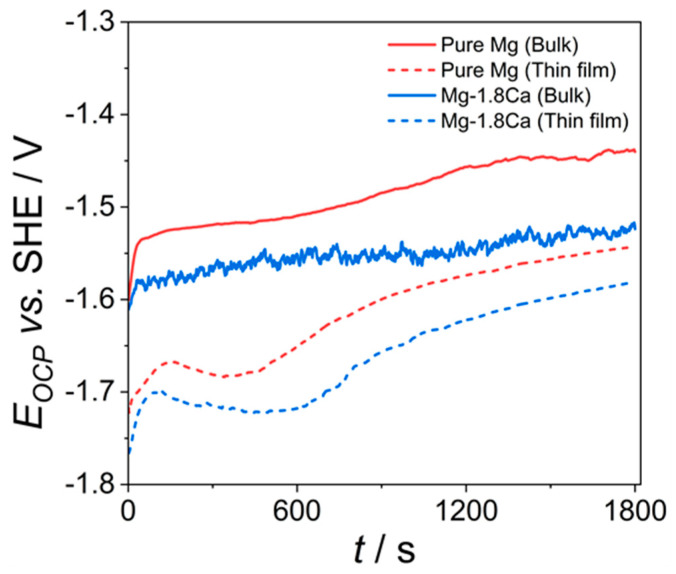
Open circuit potential (OCP) values for the bulk and thin-film samples.

Open circuit potential (OCP) values measured during immersion up to 30 min are presented in Figure 5. The bulk samples showed a rapid increase in their OCPs within the first 30 s of immersion, while this initial increase in the OCPs lasted until around 145 s and 120 s for the thin-film pure Mg and Mg-1.8Ca (at.%) samples, respectively. This suggests the formation of a passive layer, developing at a faster rate on the surface of bulk samples during the initial contact with electrolyte compared to the thin films [24]. In the following period, the OCP of the bulk pure Mg continued to increase gradually and became steady after 20 min of immersion. However, the change in the OCP of the bulk Mg-1.8Ca (at.%) alloy resulted in highly erratic behavior and a much lower increase rate over prolonged immersion time. This can be attributed to the presence of a high fraction of the Mg_2_Ca intermetallic phase, which can dissolve preferentially due to its lower nobility than Mg and thus reduce the stability of surface film [25,26]. After the initial rise in the OCPs, the thin-film samples similarly exhibited a slight decrease in their OCPs, followed by a considerable increase until the end of the test. The ranking of the OCPs remained consistent throughout the immersion period, arranged in ascending order as follows: Mg-1.8Ca (thin film) < pure Mg (thin film) < Mg-1.8Ca (bulk) < pure Mg (bulk).

**Figure 6:**
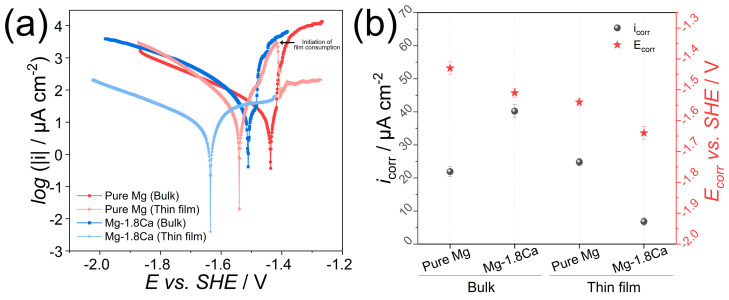
Polarization test results of the bulk and thin-film samples: (**a**) potentiodynamic polarization curves; and (**b**) i_corr_ and E_corr_ values derived from (**a**).

The potentiodynamic polarization curves of the bulk and thin-film samples and the parameters of corrosion potential (E_corr_) and corrosion current density (i_corr_) derived from the curves are illustrated in Figure 6. As stated earlier, the i_corr_ values were determined by the extrapolation of the cathodic branch of the Tafel plots to avoid the effect of abnormal anodic behavior exclusively observed in Mg (negative-difference effect) [27]. A comparison of the cathodic behaviors of the bulk samples showed that the cathodic reaction kinetics in pure Mg increased after the addition of Ca. This suggests an increase in the kinetics of the water reduction reaction through Ca addition, likely due to the presence of active cathodic areas that resulted from surface roughening. On the contrary, thin-film Mg-1.8Ca (at.%) alloy demonstrated significantly lower cathodic reaction kinetics compared to thin-film pure Mg and other bulk samples. This also resulted in the thin-film Mg-1.8Ca (at.%) alloy having the lowest i_corr_ value, i.e., corrosion rate, among all the samples, even though its bulk counterpart showed the highest i_corr_ value. On the other hand, the bulk and thin-film pure Mg samples demonstrated relatively comparable corrosion rates. Therefore, it can be inferred that the large difference in the corrosion rates of bulk and thin-film forms of the Mg-1.8Ca (at.%) alloy is primarily related to the dual microstructure (α-Mg + Mg_2_Ca) observed in the bulk sample. The addition of Ca in both bulk and thin-film samples led to a shift in the corrosion potential (E_corr_) values in the negative direction, which can be attributed to the intrinsically low standard reduction potential of Ca, further reducing the electrode potential of Mg [28,29].

The thin-film Mg-1.8Ca (at.%) alloy also exhibited exceptionally lower anodic reaction kinetics compared to the other samples, which demonstrated much faster anodic current propagation. In addition, an inflection point was observed at approximately −1.43 V (~200 mV vs. E_corr_) towards the end of the anodic branch of the thin-film Mg-1.8Ca (at.%) alloy. This indicates the formation of a stable and protective surface film layer, eventually undergoing a breakdown at a certain potential value. The other samples also displayed unobvious inflection points for the bulk pure Mg, the bulk Mg-1.8Ca (at.%) and thin-film pure Mg, which were determined as -1.42 V (~20 mV vs. E_corr_), −1.48 V (~30 mV vs. E_corr_) and −1.48 V (~60 mV vs. E_corr_), respectively. However, the proximity of these points to the corresponding E_corr_ values implied that the films formed on their surfaces had low protective properties. It is also worth noting that a sudden drop in the anodic current of thin-film pure Mg was observed after it reached its peak. Since this occurred following the breakdown of the passive layer, it can be inferred that it was linked to the consumption of the surface film rather than the characteristics of the surface oxide. Similar behavior in the anodic curve of the thin-film Mg alloys was reported elsewhere [10,30]. The Mg-1.8Ca (at.%) alloy did not exhibit thin-film consumption during the polarization test, further confirming the efficacy of the protectiveness of the surface layer on this sample and its excellent corrosion resistance.

**Figure 7:**
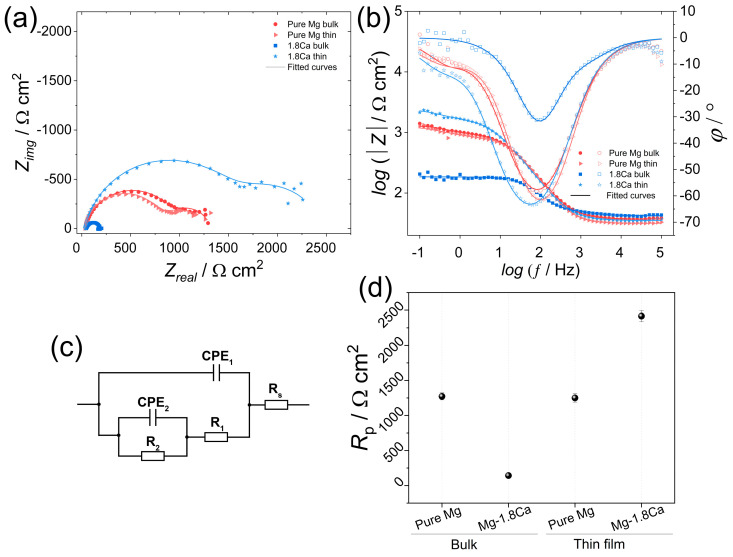
Electrochemical impedance spectroscopy (EIS) results of the bulk and thin-film samples: (**a**) Nyquist and (**b**) Bode plots; (**c**) equivalent circuit used for fitting; and (**d**) polarization resistance (Rp) values calculated from fitted EIS spectra.

Figure 7 shows the EIS spectra of the bulk and thin-film samples, together with the equivalent circuit used for fitting the spectra and the polarization resistance (R_p_) values calculated for each sample. The EIS spectra reveal that all samples displayed similar trends, mainly characterized by two-time constants with no inductive values. In the Nyquist plots, the large capacitive loops at high frequencies, corresponding to the protective properties of oxide/hydroxide surface film were followed by relatively smaller capacitive loops at low frequencies, associated with the double layer at the sample/electrolyte interface [31,32]. The EIS parameters, derived by fitting the spectra with the equivalent circuit, as shown in Figure 7c, are given in Table 2, where Rs is the solution resistance; R_1_ is the film resistance; R_2_ is the charge transfer resistance; and CPE_1_ and CPE_2_ are the constant phase elements referring to the capacitance of film and electric double layer, respectively. The R_p_ values were calculated based on the infinite impedance of capacitive components as the frequency approaches zero. As illustrated in Figure 7d, the thin-film Mg-1.8Ca (at.%) alloy exhibited, by far, the highest Rp value among the studied samples. This indicates the enhanced corrosion resistance since there is an inverse relationship between Rp and the corrosion rate according to the Stern–Geary equation [33]. In line with the potentiodynamic polarization test results, the bulk Mg-1.8Ca (at.%) alloy displayed the lowest Rp value, while the bulk and thin-film pure Mg samples showed highly comparable resistance. This is also clear in their spectra shown in Figure 7a,b, as they showed similar total resistance and phase shift, resulting in comparable charge transfer and film resistance values. It should also be noted that the maximum phase angle for the thin-film Mg-1.8Ca (at.%) alloy shifted to lower frequencies with a higher value of maximum phase angle, i.e., more negative, compared to the other samples, as illustrated in Figure 7b. This implies that a more capacitive behavior occurred mainly at low frequencies, which is typically associated with the slower process, such as charge transfer through the surface layer. Thus, the shift and increase in the maximum phase angle are likely the indication of a protective film, acting as a barrier to charge transfer. On the other hand, at high and middle frequencies, relating to faster reaction kinetics, the thin-film Mg-1.8Ca (at.%) alloy exhibited slightly lower total impedance and phase shift compared to the thin-film pure Mg. This might result from the active dissolution of less noble Ca atoms in the thin-film samples driven by its more negative standard electrode potential.

**Figure 8:**
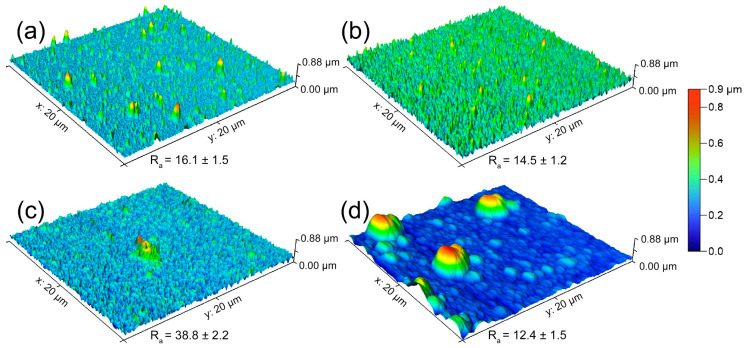
Atomic force microscopy (AFM) images of the thin-film (**a**,**c**) pure Mg and (**b**,**d**) Mg-1.8 Ca (at.%) alloy taken (**a**,**b**) before and (**c**,**d**) after immersion in 0.9% NaCl solution for 1 h.

An alloying element in Mg significantly influences the corrosion behavior of the final alloy, primarily through its ability to form solid solution or intermetallic compounds with Mg and/or other alloying elements [34,35]. These typically affect the stability and protectiveness of the surface film layer, either positively or negatively, or promote localized corrosion by creating micro galvanic couplings with matrix phase. Many intermetallic compounds generally have a higher nobility (more cathodic role) than Mg due to the low standard electrode potential of Mg [36]. However, Mg_2_Ca is one of the few elements that has a lower electrochemical potential than Mg and dissolves preferentially in place of the α-Mg matrix phase [25,26,37]. Moreover, the dissolution of Mg_2_Ca, which majorly precipitates along grain boundaries, can lead to the undermining of Mg grains, thereby significantly increasing the corrosion rate [38]. It was also demonstrated in this study that single-phase pure Mg exhibited similar and comparable corrosion performance in both bulk and thin-film form. However, this behavior differed significantly in the Mg-1.8Ca (at.%) alloy. The corrosion resistance of the bulk Mg-1.8Ca (at.%) alloy was extremely poor primarily due to the preferential dissolution of the anodic Mg2Ca intermetallic phase, which also adversely affected the stability of the surface film. On the other hand, the restricted diffusion of atoms during the thin-film production of the same alloy composition prevented the intermetallic formation, with negative effects on the corrosion resistance, like Mg_2_Ca. As shown in Figure 8, the surface topography of thin films after 1 h of immersion revealed that the structure, initially consisting of extremely thin and uniformly distributed columns, progressed to the form of deep pits in pure Mg, leading to the formation of a jagged surface. This indicates that the pure Mg thin film experienced substantial corrosion, leading to the deterioration of the initially uniform structure. Meanwhile, the Mg-1.8Ca (at.%) alloy displayed a surface form containing several large uncorroded areas but mostly covered with small and homogeneous rounded peaks. This suggests that the presence of Ca in the thin-film alloy played a crucial role in modifying the corrosion behavior. During immersion in the thin-film Mg-1.8Ca (% at.) alloy, the film layer may have protected the surface more effectively, preventing preferential corrosion progression towards the spaces between columns, thus leading to the formation of a rounded appearance.

**Figure 9:**
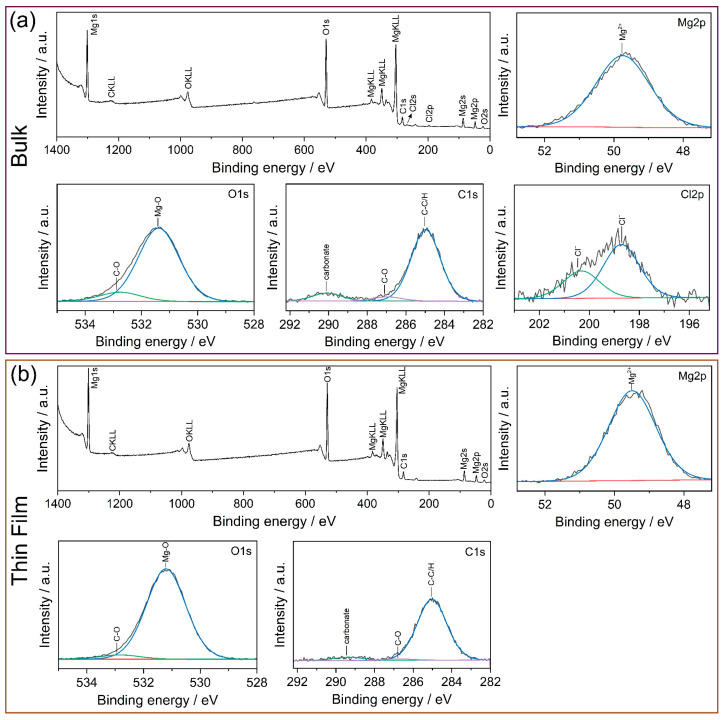
X-ray photoelectron spectroscopy (XPS) results of the (**a**) bulk and (**b**) thin-film Mg-1.8 Ca (at.%) alloy after immersion in 0.9% NaCl solution for 1 h.

XPS spectra of the bulk and thin-film Mg-1.8 Ca (at.%) alloy after immersion in 0.9% NaCl solution for 1 h are given in Figure 9. In order to evaluate the chemical state of Mg^2+^, the concept of the modified Auger parameter (MAP) was used by calculating the sum of the binding energy (BE) of Mg2p and the kinetic energy of an Auger electron line (MgKLL) [39]. The calculated value of MAP for Mg was 1230.3 eV for both bulk and thin-film samples. This value coincided well with the reference data obtained for Mg(OH)_2_, as discussed in [39,40]. Additionally, the bulk and thin-film samples showed O:H ratios of 1.8 and 1.9, respectively, which were very close to the value of 2 expected from Mg(OH)_2_. The O1s spectra for both samples can be resolved into two peaks: the main peak at ~531.4 eV can be attributed to Mg-based oxides, while the second peak at ~531.7 eV was associated with the C-O group. The C1s spectra for both samples were separated into three peaks found at ~290.0, ~286.9 and ~284.9, corresponding to C in carbonate, C-OH/C-O-C groups and contamination C, respectively. The spectra of Cl2p were only observed for the bulk Mg-1.8 Ca (at.%) alloy, while the thin-film sample did not show any peaks related to Cl. This indicates that the corrosion product form on the surface of the bulk sample showed a higher tendency to adsorb Cl^−^ ions due to its more porous nature. Furthermore, no evidence of Ca in the corrosion products was found in the XPS analyses, implying that Ca did not directly contribute to the formation of the layer of the surface film in either of the samples.

Based on the pure Mg and Mg-Ca alloys with a specific concentration in this study, it can be concluded that in order for the corrosion properties of a multi-element thin-film system designed for combinatorial analysis to be comparable to those of the bulk versions, the phases obtained in both systems should be similar or equivalent. However, the atomic diffusion limitation in thin-film production generally prevents the formation of typical intermetallic phases observed in bulk materials and can result in significant differences in corrosion resistance, as seen in this study with the Mg-1.8Ca (% at.) alloy. Therefore, in combinatorial thin-film analyses, it may be more effective and accurate to focus on alloy combinations where a single-phase structure is expected in bulk form. It is also crucial to simulate and thoroughly analyze the solubility limits of the elements with each other to ensure accurate comparisons.

## 4. Conclusions

Pure Mg and a Mg-1.8Ca (at.%) alloys in bulk and thin-film forms were successfully produced, and their corrosion behaviors were comparatively investigated. The following conclusions can be drawn based on the findings:

- The addition of 1.8 at.% Ca to pure Mg resulted in a refinement in the microstructure in both bulk and thin-film forms.
- A significant amount of Mg_2_Ca intermetallic phase was formed in the bulk Mg-1.8Ca (at.%) alloy, whereas only a single-phase (α-Mg phase) was formed in the bulk pure Mg and thin films.
- The bulk and thin-film pure Mg samples exhibited comparable corrosion rates due to the single-phase microstructure.
- The corrosion behaviors of bulk and thin-film Mg-1.8Ca (at.%) alloy significantly differed from each other; the bulk version showed the poorest corrosion performance among the studied samples due to the presence of less noble Mg_2_Ca intermetallic.
- The thin-film Mg-1.8Ca (at.%) alloy displayed a superior corrosion resistance, attributed to its single-phase microstructure and the formation of a robust protective surface film.
- Based on the findings in this study, several future research directions on the Mg-Ca thin films, such as mechanical properties under dynamic loading conditions, long-term corrosion performance for biomedical applications and/or combinatorial alloy developments, can be proposed.

## Figures and Tables

**Table 1:** Lattice constants and *c*/*a* ratios of pure Mg and Mg-1.8 Ca (at.%) alloys.

Condition	Sample	*a*/nm	*c*/nm	*c*/*a*
-	Pure Mg *	0.32094	0.52112	1.62373
Bulk	Pure Mg	0.32823	0.53214	1.62123
	Mg-1.8Ca	0.32714	0.53199	1.62618
Thin film	Pure Mg	0.30714	0.50098	1.62373
	Mg-1.8Ca	0.30714	0.50045	1.62938

* Values from JCPDS #35-0821.

**Table 2:** Electrochemical parameters derived from EIS measurements.

Condition	Sample	R_s_ /Ω cm^2^	R_1_ /Ω cm^2^	R_2_ /Ω cm^2^	CPE_1_ /Ω^−1^ s^n^ cm^−2^ 10^−5^	n_1_	CPE_2_ /Ω^−1^ s^n^ cm^−2^ 10^−3^	n_2_
Bulk	Pure Mg	37.1	947	325	2.35	0.87	1.26	0.92
	Mg-1.8Ca	42.1	22	121	2.85	0.86	0.02	0.96
Thin film	Pure Mg	31.7	795	455	1.68	0.92	1.31	0.76
	Mg-1.8Ca	35.1	1664	752	2.47	0.88	0.88	0.86

## Data Availability

The original contributions presented in this study are included in the article. Further inquiries can be directed to the corresponding author.

## References

[1] Zeng Z., Stanford N., Davies C.H.J., Nie J.-F., Birbilis N. (2018). Magnesium Extrusion Alloys: A Review of Developments and Prospects. Int. Mater. Rev..

[2] Rout P.K., Roy S., Ganguly S., Rathore D.K. (2022). A Review on Properties of Magnesium-Based Alloys for Biomedical Applications. Biomed. Phys. Eng. Express.

[3] Bairagi D., Mandal S. (2022). A Comprehensive Review on Biocompatible Mg-Based Alloys as Temporary Orthopaedic Implants: Current Status, Challenges, and Future Prospects. J. Magnes. Alloys.

[4] Zengin H., Hassel A.W. (2025). Magnesium Alloys with Rare Earth Elements—A Review of the Recent Progress on the Corrosion Properties. Corros. Sci..

[5] Liu R.L., Scully J.R., Williams G., Birbilis N. (2018). Reducing the Corrosion Rate of Magnesium via Microalloying Additions of Group 14 and 15 Elements. Electrochim. Acta.

[6] Recktenwald D., Mardare C.C., Mardare A.I., Jinga L.-I., Socol G., Hassel A.W. (2020). Combinatorial Screening of Dysprosium-Magnesium-Zinc Alloys for Bioresorptive Implants. Electrochim. Acta.

[7] Ludwig A. (2019). Discovery of New Materials Using Combinatorial Synthesis and High-Throughput Characterization of Thin-Film Materials Libraries Combined with Computational Methods. Npj Comput. Mater..

[8] Mao S.S. (2013). High Throughput Growth and Characterization of Thin Film Materials. J. Cryst. Growth.

[9] Klemm S.O., Schauer J.-C., Schuhmacher B., Hassel A.W. (2011). High Throughput Electrochemical Screening and Dissolution Monitoring of Mg–Zn Material Libraries. Electrochim. Acta.

[10] Zengin H., Mardare A.I., Popescu-Pelin G., Socol G., Hassel A.W. (2024). Magnesium-Calcium Thin Films at Low Calcium Concentrations for Anode Materials in Biodegradable Implantable Primary Batteries. Mater. Lett..

[11] Hans M., Keuter P., Saksena A., Sälker J.A., Momma M., Springer H., Nowak J., Zander D., Primetzhofer D., Schneider J.M. (2021). Opportunities of Combinatorial Thin Film Materials Design for the Sustainable Development of Magnesium-Based Alloys. Sci. Rep..

[12] Keuter P., to Baben M., Aliramaji S., Schneider J.M. (2023). CALPHAD-Based Modelling of the Temperature–Composition–Structure Relationship during Physical Vapor Deposition of Mg-Ca Thin Films. Materials.

[13] Zuo Y.B., Fu X., Mou D., Zhu Q.F., Li L., Cui J.Z. (2015). Study on the Role of Ca in the Grain Refinement of Mg–Ca Binary Alloys. Mater. Res. Innov..

[14] Ali Y., Qiu D., Jiang B., Pan F., Zhang M.-X. (2015). Current Research Progress in Grain Refinement of Cast Magnesium Alloys: A Review Article. J. Alloys Compd..

[15] Kulyasova O.B., Khudododova G.D., Dyakonov G.S., Zheng Y., Valiev R.Z. (2022). Effect of Microstructure Refinement on the Corrosion Behavior of the Bioresorbable Mg-1Zn-0.2Ca and Mg-1Ca Alloys. Materials.

[16] Xie Z.-R., Zhang C., Pan H.-C., Wang Y.-X., Ren Y.-P., Qin G.-W. (2023). Microstructures and Bio-Corrosion Resistances of as-Extruded Mg–Ca Alloys with Ultra-Fine Grain Size. Rare Met..

[17] Nayeb-Hashemi A.A., Clark J.B. (1988). Phase Diagrams of Binary Magnesium Alloys.

[18] Seong J.W., Kim W.J. (2015). Development of Biodegradable Mg–Ca Alloy Sheets with Enhanced Strength and Corrosion Properties through the Refinement and Uniform Dispersion of the Mg2Ca Phase by High-Ratio Differential Speed Rolling. Acta Biomater..

[19] Zhang Y., Feng X., Huang Q., Li Y., Yang Y. (2023). Enhancing Mechanical Properties and Degradation Performance of Mg−0.8wt.%Ca Alloy by Directional Solidification. Trans. Nonferrous Met. Soc. China.

[20] Kirkland N.T., Birbilis N., Walker J., Woodfield T., Dias G.J., Staiger M.P. (2010). In-Vitro Dissolution of Magnesium–Calcium Binary Alloys: Clarifying the Unique Role of Calcium Additions in Bioresorbable Magnesium Implant Alloys. J. Biomed. Mater. Res. B Appl. Biomater..

[21] Wang D., Jiang W., Li S., Yan X., Wu S., Qiu H., Guo S., Zhu B. (2023). A Comprehensive Review on Combinatorial Film via High-Throughput Techniques. Materials.

[22] Su W.B., Chang C.S., Tsong T.T. (2009). Quantum Size Effect on Ultra-Thin Metallic Films. J. Phys. Appl. Phys..

[23] Chason E., Sheldon B.W., Freund L.B., Floro J.A., Hearne S.J. (2002). Origin of Compressive Residual Stress in Polycrystalline Thin Films. Phys. Rev. Lett..

[24] Ding H.-Y., Li H., Wang G.-Q., Liu T., Zhou G.-H. (2018). Bio-Corrosion Behavior of Ceramic Coatings Containing Hydroxyapatite on Mg-Zn-Ca Magnesium Alloy. Appl. Sci..

[25] Gnedenkov A.S., Sinebryukhov S.L., Filonina V.S., Egorkin V.S., Ustinov A.Y., Sergienko V.I., Gnedenkov S.V. (2022). The Detailed Corrosion Performance of Bioresorbable Mg-0.8Ca Alloy in Physiological Solutions. J. Magnes. Alloys.

[26] Bahmani A., Arthanari S., Shin K.S. (2019). Corrosion Behavior of Mg–Mn–Ca Alloy: Influences of Al, Sn and Zn. J. Magnes. Alloys.

[27] Song G., Atrens A., Dargusch M. (1998). Influence of Microstructure on the Corrosion of Diecast AZ91D. Corros. Sci..

[28] Haynes W.M. (2015). CRC Handbook of Chemistry and Physics.

[29] Zengin H., Hofinger M., Silva Campos M.D.R., Blawert C., Polat S., Nienaber M., Bohlen J., Zheludkevich M., Hassel A.W. (2025). Corrosion Behaviour of Biodegradable Mg-Zn-Mn-Ce, Mg-Zn-Ca-Ce, and Mg-Zn-Ca-Mn Quaternary Magnesium Alloys in Phosphate-Buffered Saline. J. Alloys Compd..

[30] Khan M.M., Rahman Z.U., Deen K.M., Shabib I., Haider W. (2020). Sputtered Mg100-xZnx (0 ≤ x ≤ 100) Systems as Anode Materials for a Biodegradable Battery Aimed for Transient Bioelectronics. Electrochim. Acta.

[31] Wang L., Snihirova D., Deng M., Wang C., Vaghefinazari B., Wiese G., Langridge M., Höche D., Lamaka S.V., Zheludkevich M.L. (2021). Insight into Physical Interpretation of High Frequency Time Constant in Electrochemical Impedance Spectra of Mg. Corros. Sci..

[32] Zengin H., Ari S., Turan M.E., Hassel A.W. (2023). Evolution of Microstructure, Mechanical Properties, and Corrosion Resistance of Mg–2.2Gd–2.2Zn–0.2Ca (Wt%) Alloy by Extrusion at Various Temperatures. Materials.

[33] Stern M., Geary A.L. (1957). Electrochemical Polarization: I. A Theoretical Analysis of the Shape of Polarization Curves. J. Electrochem. Soc..

[34] Gusieva K., Davies C.H.J., Scully J.R., Birbilis N. (2015). Corrosion of Magnesium Alloys: The Role of Alloying. Int. Mater. Rev..

[35] Cao F., Song G.-L., Atrens A. (2016). Corrosion and Passivation of Magnesium Alloys. Corros. Sci..

[36] Hort N., Huang Y., Kainer K.U. (2006). Intermetallics in Magnesium Alloys. Adv. Eng. Mater..

[37] Xu C., Wang J., Chen C., Wang C., Sun Y., Zhu S., Guan S. (2023). Initial Micro-Galvanic Corrosion Behavior between Mg_2_Ca and α-Mg via Quasi-in Situ SEM Approach and First-Principles Calculation. J. Magnes. Alloys.

[38] Jeong Y.S., Kim W.J. (2014). Enhancement of Mechanical Properties and Corrosion Resistance of Mg–Ca Alloys through Microstructural Refinement by Indirect Extrusion. Corros. Sci..

[39] Duchoslav J., Truglas T., Groiß H., Riener C.K., Arndt M., Stellnberger K.H., Luckeneder G., Angeli G., Stifter D. (2019). Structure and Chemistry of Surface Oxides on ZnMgAl Corrosion Protection Coatings with Varying Alloy Composition. Surf. Coat. Technol..

[40] Duchoslav J., Steinberger R., Arndt M., Keppert T., Luckeneder G., Stellnberger K.H., Hagler J., Angeli G., Riener C.K., Stifter D. (2015). Evolution of the Surface Chemistry of Hot Dip Galvanized Zn–Mg–Al and Zn Coatings on Steel during Short Term Exposure to Sodium Chloride Containing Environments. Corros. Sci..

